# Dung beetle species introductions: when an ecosystem service provider transforms into an invasive species

**DOI:** 10.7717/peerj.9872

**Published:** 2020-09-25

**Authors:** Min R. Pokhrel, Stuart C. Cairns, Nigel R. Andrew

**Affiliations:** 1 Insect Ecology Lab, Natural History Museum, University of New England, Armidale, NSW, Australia; 2 Faculty of Agriculture, Agriculture and Forestry University, Bharatpur, Nepal; 3 Centre for Behavioural and Physiological Ecology, University of New England, Armidale, NSW, Australia

**Keywords:** Dung beetle, Introductions, Invasive species, Native, Introduced

## Abstract

Dung beetle introduction programmes were designed to accelerate exotic livestock dung degradation and to control dung breeding pestiferous flies and livestock parasites. The introduction programmes provided exotic dung beetle species with an opportunity to cross natural barriers and spread beyond their native range. There are no reports that explain what probable adaptation mechanisms enable particular dung beetle species to be the most successful invader. Here we identify the morphological, biological, physiological, ecological and behavioural attributes of the four most widespread and successful dung beetle species in introduced areas on a global scale in relation to the assumption that these species are different from other exotic and native dung beetles. We have recognised *Digitonthophagus gazella* (Fabricius), *Onthophagus taurus* (Schreber), *Euoniticellus intermedius* (Reiche) and *Aphodius fimetarius* (Linnaeus) as the most successful invaders based on their spread, predominance, distribution range and the reports of invasion. Each of these four species has different natural history traits that increase their fitness making them successful invaders. *D. gazella* has high fecundity and spreading ability, can instantly locate and colonise fresh and nutritious dung, and has a broad thermal window. *O. taurus* has morphological plasticity, high fecundity, high brood survival rate due to bi-parenting, and is adapted to extreme thermal and moisture conditions. *E. intermedius* has remnant-dung feeding abilities, a wide thermal window, functioning best at upper-temperature levels, and successful breeding and survival abilities at extremely low soil moisture conditions. *A. fimetarius* is small-sized, has high breeding and dispersal abilities, and is adapted to lower thermal and upper moisture extremes and variable soil conditions. Discussed here are perspectives on adaptive attributes of dung beetle species that are important to consider during their selection for redistributions. We have elaborated on the fitness and success characteristics of the four species individually. Further, we recommend a prior-introduction baseline monitoring of native dung beetle assemblages so as to evaluate the future impact of exotic dung beetle introductions on the recipient ecosystem.

## Introduction

The introduction of exotic plant and animal species for food and services has become a ubiquitous aspect of modern agriculture. To be successful, an exotic species has to cross a series of barriers or filters from an introduction event through to the subsequent stages of establishment in a new environment (Colautti & MacIsaac, 2004; Flanagan, 1991). First, human-assisted introductions into new areas enable the populations of species to cross geographical barriers and subsequently establish adult populations (Funasaki et al., 1988; Richardson et al., 2000). Second, the propagule pressure, niche requirements and community interactions determine establishment success (Colautti & MacIsaac, 2004; Duncan, 2016). Third, an established species can overcome abiotic and biotic barriers of survival, reproduce regularly, and maintain its populations is known as naturalised (Colautti & MacIsaac, 2004; Foxcroft, Pickett & Cadenasso, 2011). Fourth, any naturalised species that can produce enough offspring to sustain exotic populations and spread to new areas, is known as being invasive (Crowley et al., 2017; Foxcroft, Pickett & Cadenasso, 2011; Richardson et al., 2000). Invasion is thus a climax of consecutive stages such as human-assisted arrival, establishment, naturalisation, dispersal and multiplication (Colautti & MacIsaac, 2004; Richardson et al., 2000).

Invasive species cause environmental and economic impacts (Davis & Thompson, 2000; Richardson et al., 2000). However, the impact assessment is subjective and needs to take into consideration the complexity of direct and indirect interactions between species (Daehler, 2001). The impact can be assessed as being minimal, moderate or substantial. In relation to this it could be categorised as being beneficial, neutral or harmful (Daehler, 2001). Various frameworks (Colautti & MacIsaac, 2004), indicators (Davis & Thompson, 2000) and ecological definitions (Daehler, 2001; Hill et al., 2016) have been proposed in describing invasion. Based on their levels of impact (bio-pollution) on their new environment, community or ecosystem, species can be differentiated as being ‘potentially invasive’ or ‘invasive’ (Zaiko et al., 2010).

Insect species are deliberately introduced as biological control agents, pollinators, nutrient recyclers and food and fibre producers (Bornemissza, 1976; Davis, 1960; Roy & Wajnberg, 2007). It is sometimes unpredictable as to which exotic insect species might be invasive outside of its native range and cross barriers to become a transnational species (De Oca & Halffter, 1998; Filho et al., 2018). For instance, the Asian harlequin ladybird beetle, *Harmonia axyridis* (Pallas), once introduced into North America and Europe as an aphid predator, is now recognised as a notorious invader in the United Kingdom (UK), that has such negative effects as threatening native predators, and becoming a crop pest and human nuisance (Roy & Wajnberg, 2007). The large garden bumblebee, *Bombus ruderatus* (Fabricius), introduced into Chile for crop pollination has caused the severe decline and local extinction of native bumblebees and pollinators throughout South America (Aizen et al., 2018). The large earth bumblebee, *Bombus terrestris* (Linnaeus), a commercial greenhouse and open-air crop pollinator, was accidentally introduced into the island state of Tasmania, Australia (Dafni et al., 2010; Semmens, Turner & Buttermore, 1993). It is common and widespread throughout the island state and has been found visiting 156 introduced and 14 native plant species (Hingston, 2007; Semmens, 1996). *B. terrestris* has impacted upon natural communities through increasing the pollination success of invasive plant species such as the nitrogen-fixing, *Lupinus arboreus* Sims and competitively excluded the European honeybee, *Apis mellifera* Linnaeus from its pollination (Hingston, 2007; Stout, Kells & Goulson, 2002). Broadly, *B. terrestris* has reduced reproductive success in native plants and outcompeted native pollinators for pollen and nectar (Dafni et al., 2010; Hingston, 2007; Stout & Morales, 2009). Australia has recently listed 17 insect species as invasive that include nine ant, three wasp, four bee and one beetle species—based on the harm they caused to environment. These are examples of non-native insects that became deleterious to the environment and negatively impact on ecosystem functioning, but are not considered as being agricultural pests (McGeoch, 2020).

The impact of invasive species is regarded as being direct if they prey upon, parasitise hosts or cause diseases (Comont & Roy, 2011) and as being indirect if they compete with native or naturalised species, and have a broad impact on the invaded ecosystem (Hulme & Vilà, 2017). Dung beetle species do not have any direct impacts through predation, parasitism, or by causing diseases to other taxa (Bornemissza, 1979; Dymock, 1993; Mackereth et al., 2013). Therefore, the impact of introduced dung beetle species is considered indirect usually because they compete with native or resident dung beetle species and can lead to change in their assemblage (Blanco et al., 2013; Filho et al., 2018; Tyndale-Biscoe, 1990). The objective assessment of the environmental and economic impacts of any invasive species needs a comprehensive method (Ricciardi et al., 2017; Simberloff et al., 2013; Simberloff & Stiling, 1996). The assessment of invasive dung beetle species impact requires rigorous research data on native dung beetle assemblages, dung resources and the trophic structure of dung dependent species (Belnap et al., 2012; Hulme & Vilà, 2017). Further, the impact assessment approach for undisturbed habitats and disturbed agricultural habitats should be different (Goosey et al., 2019; King & Tschinkel, 2008). The impact on human-made novel ecosystems, such as improved pasturelands and native grasslands may be assessed based on ecosystem services and economic benefits rather than only biodiversity and conservation perspectives (Beynon, Wainwright & Christie, 2015; Nichols et al., 2008).

Generally, invasive species may show some or all of the following characteristics. They reproduce more frequently and achieve high abundances, have short generation times, mature early, disperse quickly, are generalist feeders and tolerate habitat stressors (Comont & Roy, 2011; Davidson, Jennions & Nicotra, 2011; Nyamukondiwa, Kleynhans & Terblanche, 2010; Roy et al., 2008). Overwintering is advantageous from the perspective of increasing fitness and survival in the face of unfavourable temperature fluctuations (Pullin, 1996). The invasive dung beetle, *D. gazella* successfully overwinters, but its survival is affected by soil type (Fincher & Hunter, 1989). The European fire ant, *Myrmica rubra* Linnaeus, an invasive species in parts of North America and Asia, forms multi-nest, multi-queen colonies and prefers habitats that are unsuitable for native ants (Goodman & Warren II, 2019). Invasive species show greater plasticity across a range of morphological, biological, physiological and fitness traits compared to the co-occurring non-invasive species (Davidson, Jennions & Nicotra, 2011; Nyamukondiwa, Kleynhans & Terblanche, 2010). For example the invasive Asian harlequin ladybird beetle shows melanic morphs that vary with seasons and environment conditions (Michie et al., 2010). Melanism in the peppered moth, *Biston betularia* (Linnaeus), is cryptic against predatory birds and also a form of thermal melanism that enables more heat absorption (True, 2003). Greater morphological plasticity in the invasive honeysuckle, *Lonicera japonica* Thunberg, a vine species introduced to the USA, enables it to be a super invader (Schweitzer & Larson, 1999). Similarly, plasticity in growth and reproduction of the South African daisy, *Senecio pterophorus* DC, increases fitness and adapts to high levels of disturbance and stressors (Caño et al., 2008). Phenotypic plasticity in the invasive springtail, *Pogonognathellus flavescens* Tullberg, enhances fitness under desiccated conditions (Chown et al., 2007). Phenotypic plasticity also contributes to acute low-temperature tolerances in the invasive Mediterranean fruit fly, *Ceratitis capitata* Wiedemann (Nyamukondiwa, Kleynhans & Terblanche, 2010). Indeed, there are many other examples that illustrate invasive species have enhanced phenotypic plasticity than non-invasive species (Davidson, Jennions & Nicotra, 2011).

In this article, we review the natural history and adaptation traits that potentially led to the success of dung beetles as invasive species. We conducted this review as there are no reports that explain what natural history traits and adaptation attributes that have contributed to these four dung beetle species to invade different geographic ranges, spread globally and become successful invaders or invasive species. As part of this, we highlight the potential invasion risks of exotic dung beetle species and recommend assessment of dung beetle traits in designing dung beetle redistribution programmes. We hope that this article will provide critical information to researchers, policy makers and livestock growers in the countries where both dung beetle species have been introduced, and the introduction programmes are ongoing.

## Survey Methods

We conducted a systematic search for available peer-reviewed publications from 1990 to 2020 and selected grey literature and articles published between 1940 and 1989. We searched Google Scholar, Scopus, Web of Science and Research Gate electronic databases by using various search expressions. We searched using the terms: ‘invasive AND dung beetle’, ‘dung beetle AND introduction’, ‘dung beetle AND competition’ as multiple search expressions. Since the following four dung beetle species were known as being invasive, we also searched: ‘invasive AND *Onthophagus gazella*’, ‘invasive AND *Onthophagus taurus’, ‘*invasive AND *Euoniticellus intermedius*’, ‘invasive AND *Aphodius fimetarius*’. Further, we searched species names to obtain information on the traits and attributes of these species. We applied search to the article titles, abstracts and keywords in such a way that most articles of our interest were included. We included literature from the cited references that describe the traits and attributes of invasive dung beetles. To interpret the broader prospects of invasion ecology, we included articles from cited references and the grey literature. We excluded literature-based on title and abstract information—that were duplicates, in languages other than English and with a focus different from the area of our interest. Lastly, we acknowledge that the literature might not be a complete list, but we consider our study is representative and comprehensive.

## Invasive Dung Beetle Species

Dung beetle taxa consist of approximately 9,500 species from the families Scarabaeidae (Sacarbaeinae and Aphodiinae subfamilies) and Geotrupidae (Cabrero-Sañudo & Lobo, 2009; Davis & Scholtz, 2001). A total of 39 dung beetle species have become established in Australia, New Zealand and the USA having been deliberately introduced to breakdown dung, to control pestiferous flies and livestock helminth parasites (Bornemissza, 1976; Dymock, 1993; Edwards, 2007; Funasaki et al., 1988). This includes 25 species in Australia, one in New Zealand and 23 in the USA, and the species were introduced from Africa, Asia and Europe (Blank, Blank & Olson, 1983; Edwards, 2007; Funasaki et al., 1988). In addition, 49 accidentally introduced dung beetle species are reportedly established in these three countries which includes 22 species in Australia, 14 in New Zealand and 22 in the USA (Brown, 1940; Emberson & Matthews, 1973; Stebnicka, 2001, 2009). Human-assisted (deliberate and accidental) introductions have enabled dung beetles to cross the geographical barriers and spread globally and the species are recognised as being invasive on the basis of their distribution, success in a new environment, their impacts on native species and the invaded ecosystem (Colautti & MacIsaac, 2004; Davis & Thompson, 2000; Strayer, 2012). Out of those species actively dispersing, four dung beetle species namely–*Digitonthophagus gazella* (Fabricius) *Onthophagus taurus* Schereber, *Euoniticellus intermedius* (Reiche), and *Aphodius fimetarius* (Linnaeus) are continuously spreading to every continent on Earth, except Antarctica (Filho et al., 2018; Floate et al., 2017; Kryger et al., 2006; Miraldo et al., 2014). These dung beetle species are explosive in terms of multiplication, spread, competition and abundance and have expanded their distributions, and reported being invasive (Bertone et al., 2005; Bornemissza, 1976; Bornemissza, 1979; De Oca & Halffter, 1998; Filho et al., 2018; Floate et al., 2017; Medina, 2016; Silva et al., 2016; Stebnicka, 2009).

### *Digitonthophagus* *gazella* (previously *Onthophagus gazella*)

**Figure 1:**
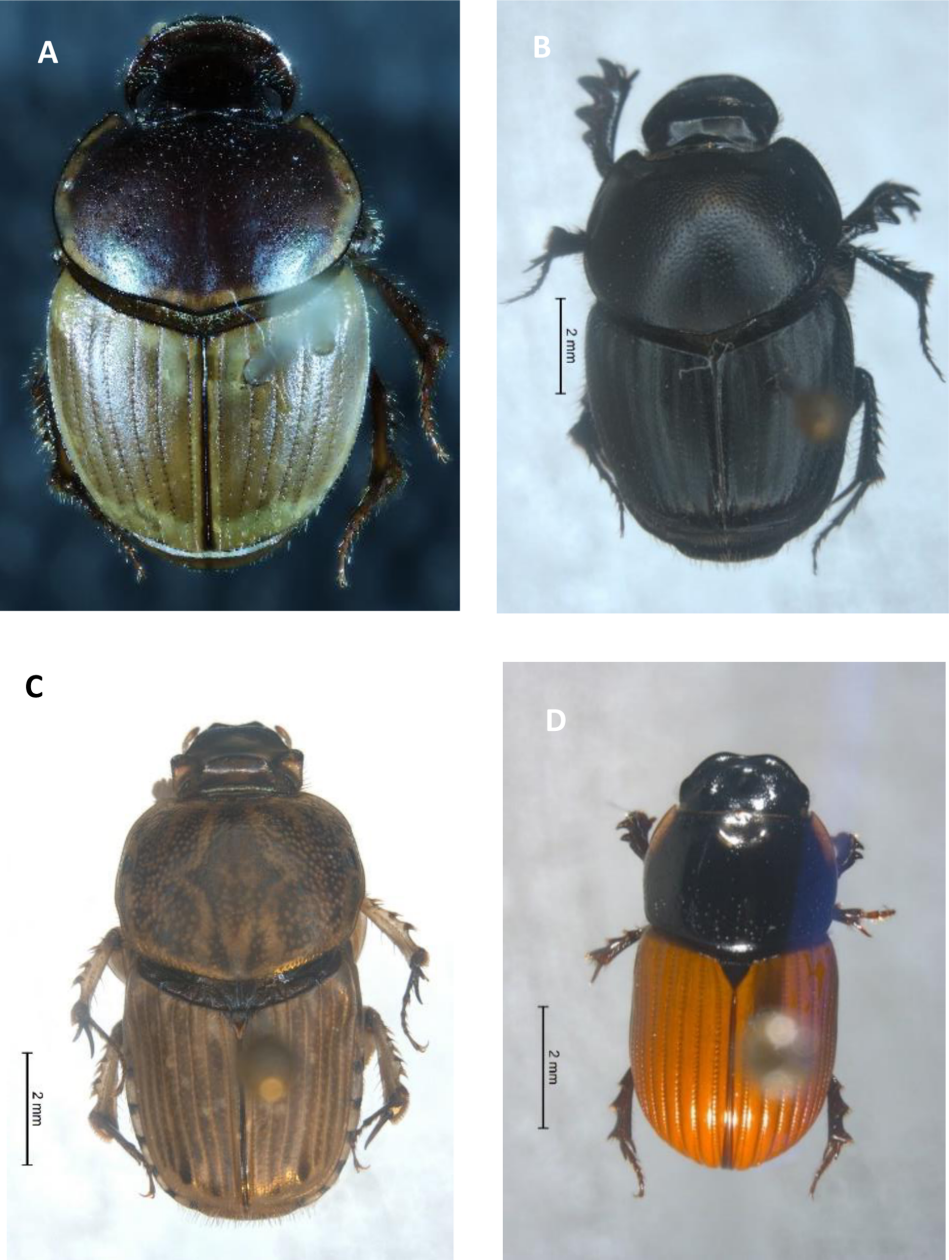
Invasive dung beetle species. (A) *Digitonthophagus gazella*, (B) *Onthophagus taurus*, (C) *Euoniticellus intermedius*, (D) *Aphodius fimetarius*. Photographs by Min Raj Pokhrel.

*Digitonthophagus gazella* (Fig. 1) is native to hotter and drier Afro-Asian region (South of Sahara, and ranging into Madagascar, Asia Minor, India and Ceylon) and has spread globally (Génier & Krell, 2017; Genier & Moretto, 2017). It was first introduced into Australia in 1968 for the burial of dung, to control dung breeding pests and parasites and was the most popular species for its dung burial and removal efficiency (Bornemissza, 1976). From Australia, it was introduced into Hawaii in 1970 to control horn fly, *Haematobia irritans irritans* (Linnaeus), (Blume, Matter & Eschle, 1973) and from Hawaii, it was redistributed to the northern part on the Atlantic coast of the USA (Bertone et al., 2005; Blume & Aga, 1975). From the release sites in the USA, it radiated to other states and reached its predicted maximum distribution (Floate et al., 2017). Outside of the USA, *D. gazella* has spread to South America, colonising areas of Mexico, Colombia and Peru (Blanco et al., 2013; De Oca & Halffter, 1998; Filho et al., 2018; Noriega et al., 2010). Models suggested *D. gazella* has reached its predicted maximum distribution from about 45° N to 45° S (Floate et al., 2017). It has expanded at a rate of 50–80 km yr^−1^ in Australia (Bornemissza, 1976), 58–89 km yr^−1^ in the USA (Barbero & López-Guerrero, 1992; Hanski & Cambefort, 1991), and 42–808 km yr^−1^ in Mexico (De Oca & Halffter, 1998). The large cattle dung resources in these invaded countries support their faster spread (Kohlmann, 1994). In addition, its wider climatic tolerance may increase its survival and fitness (Montes de Oca & Halffter, 1995).

**Figure 2:**
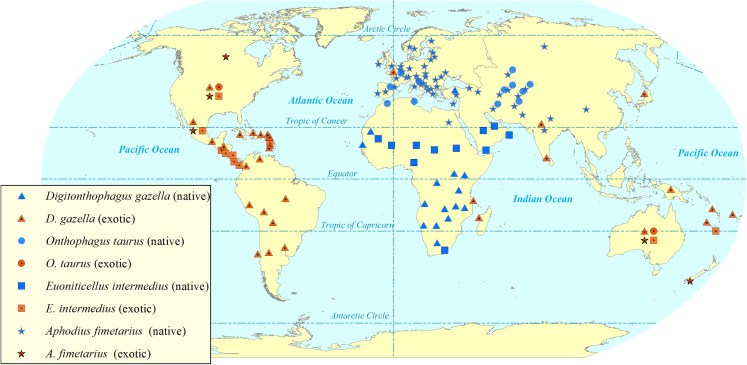
Map showing the global (native and exotic) distribution of four invasive dung beetle species. Symbols represent occurrence in a country, not the within-country distribution. Dung beetle distribution map by Sushil Lamichhane.

*Digitonthophagus gazella* is one of the most widely reported invasive dung beetle species in the world (De Oca & Halffter, 1998; Filho et al., 2018; Genier & Davis, 2017; Kohlmann, 1994; Medina, 2016; Vidaurre, Noriega & Ledezma, 2008). Its invasion has reduced native dung beetle diversity by some 40% during a period of 26 years in areas of Brazil (Filho et al., 2018). However, they have used black-light flight-intercept traps to capture dung beetles weekly only from dusk to dawn (Filho et al., 2018). Six native dung beetle species are reported to be locally extinct, and other native dung beetle species have declined their abundance (Filho et al., 2018). The invasion has changed native dung beetle communities as it outcompeted the native tunneller species—as their nesting behaviour and phenology overlap with *D. gazella*—resulting the appearance of new native dweller species (Filho et al., 2018). Tunnellers, such as *D. gazella*, dig tunnels beneath the dung pats and store the dung balls into the nest whilst, the dwellers just feed, make broods and reproduce just within the dung pats (Bornemissza, 1976). *D. gazella* crossed geographical barriers and extended its potential range of distribution (Noriega, 2002; Noriega et al., 2010; Vidaurre, Noriega & Ledezma, 2008). *D. gazella* dispersed naturally to unintended countries south of the USA such as Mexico (De Oca & Halffter, 1998; Favila, 2012; Kohlmann, 1994), Colombia (Blanco et al., 2013; Noriega, 2002) and Peru (Noriega et al., 2010). The present distribution and spread (Fig. 2) show *D. gazella* is an invasive dung beetle species.

*Digitonthophagus gazella* shows melanic and morphological variability among individuals and populations: indicating that it is a species complex (Genier & Moretto, 2017). This species was recognised as a single species and is currently separated into 14 species (Genier & Moretto, 2017). *Digitonthophagus gazella* is sexually dimorphic. The males have a wide-ranging size from horned, well-developed major males through to a minor hornless male that resembles the female (Moczek, 2011). Historically some females and dimorphic males have been described as different species resulting in confused and incorrect nomenclature (Genier & Davis, 2017; Génier & Krell, 2017; Genier & Moretto, 2017). Perhaps because of this phenotypic variation, *D. gazella* is one of the most widespread dung beetle taxa in tropical, subtropical and warm temperate regions (Genier & Moretto, 2017).

*Digitonthophagus gazella* females produce some 17 progeny within the first 20 days of adult life and can produce 90 progeny over their 60 day lifetime in laboratory conditions (Blume & Aga, 1975). Egg-to-adult development completes in 20 days at 32 °C, in 30 days at 29 °C and 52 days at 22 °C and adult emergence does not occur below 20 °C (Blume & Aga, 1975; Floate et al., 2015).

The growth-limiting low and high temperatures are 18 °C and 34 °C, and the lower and upper optimal growth temperatures are 25 °C and 30 °C, respectively (Floate et al., 2015). The limiting low and high soil moistures are 5% and 200% and the optimal lower and upper soil moistures are 35% and 100%, respectively (Floate et al., 2017) indicating that *D. gazella* survives under a wide range of soil moistures from almost wilting point to stagnant water conditions. Its high fecundity, fast multiplication and efficient dung burying ability ensure the success of future generations (Bornemissza, 1970; Doube, 1991; Young, 2007).

*Digitonthophagus gazella* is an open habitat specialist and rapidly colonises grassy pastures (De Oca & Halffter, 1998) but, it has also been reported from the lowland rain forest and tropical deciduous forest in Mexico (Thomas, 1993). Its adaptation to both open and closed habitats can have adverse effects on native dung beetles and other assemblages in the invaded ecosystems (Belnap et al., 2012; Boivin et al., 2016; Filho et al., 2018). For instance, eight native frog species, not previously reported as dung beetle predators, are now major predator of *D. gazella* in Colombia. This could be in response to change in frog prey assemblages due to presence of *D. gazella* (Blanco et al., 2013).

### Onthophagus taurus

*Onthophagus taurus* (Fig. 1) is native to central and southern Europe and Asia Minor, ranging from Spain through to Morocco, Turkey and Iran (Tyndale-Biscoe, 1990). It was successfully introduced into Australia in 1974 and was a popular species in redistribution programmes (Bornemissza, 1976) and has become widespread, becoming the most abundant exotic species in south-western Australia within two years of its release (Ridsdill-Smith, 1979). *O. taurus*, has outcompeted and replaced *O. binodis* Thunberg in its early years of establishment, which was an earlier established introduced dung beetle species in many parts of Australia (Ridsdill-Smith & Edwards, 2011). In the American context, *O. taurus* is an accidental introduction in eastern United States and is a deliberate introduction in western United States (Fincher, Stewart & Hunter, 1983; Fincher & Woodruff, 1975). Well before it was deliberately introduced, it was recorded from Florida in 1971 and later from the northeast United States (Fincher & Woodruff, 1975). The population size and trend of spread show that *O. taurus* had entered from the coastal locality of Florida panhandle (Fincher & Woodruff, 1975). Its distribution has expanded significantly within a short period from its entry location (Bertone et al., 2005; Floate et al., 2015; Floate et al., 2017; Silva et al., 2016). Modelling has suggested that large regions of central USA are suitable for the establishment of *O. taurus* (Floate et al., 2017). *O. taurus* if not detected in the USA, in the regions similar to its native range, might be due to its dispersal barriers or is undetected in its presence (Floate et al., 2017). The present distribution and spread show *O. taurus* as an invasive species.

*Onthophagus taurus* exhibits significant variations in the body colour, shape and size (especially horns and fore-tibia). Its body parts characteristics are evolutionarily labile and were in response to macro–habitats (Parzer, David Polly & Moczek, 2018; Yates et al., 2014). Only major males develop horns with the size of these horn varying with nutrition and juvenile hormone levels in larval stages (Casasa & Moczek, 2018; Emlen & Nijhout, 2001). Isolated populations of *O. taurus* have shown divergence in the shape of fore–tibiae, which relates to soil compactness and correlated with digging and nesting depths (Macagno, Moczek & Pizzo, 2016). The plasticity in body colour, shape and size increases competitive strength in dung beetle communities (Hernandez, Monteiro & Favila, 2011).

*Onthophagus taurus* exhibits a suite of reproductive behaviour such as biparental brood care (Hunt & Simmons, 1998; Hunt & Simmons, 2002), sexual polygyny (Moczek, 2011) and post-copulation sperm competition (House & Simmons, 2006). Aggressive horned (major) males safeguard and cooperate females-but sneaky hornless (minor) males never do- in making brood balls (Hunt & Simmons, 1998; Moczek & Emlen, 2000). However, both major and minor males have equal advantages to access and mate females as the minor males invest more in sperm ejaculates and sneak copulations (Moczek & Emlen, 2000; Simmons, Tomkins & Hunt, 1998). The females paired with major males produce more and larger broods than paired with minor males (Hunt & Simmons, 1998). They also can assess the quality of dung while provisioning food for offspring (Moczek, 1998). The complexes of sexual polygamy, post copulation sperm selection and biparenting improve progeny fitness through continuous sexual selection, competition and nourishment (House & Simmons, 2006; Simmons & Garcia-Gonzalez, 2008).

*Onthophagus taurus* adults emerge in 25 days at 30 °C and 105 days at 16 °C, while no emergence occurs at or below 14 °C (Floate et al., 2015). The larvae can overwinter and pupation and emergence occur with the occurrence of warmer temperature. The species has lower and upper growth limiting temperatures of 14 °C and 32 °C and lower and upper optimal growth temperatures of 16 °C and 23 °C (Floate et al., 2015). *O. taurus* has shown poor reproductive success and overwintering survival that limits its distribution in extremely cold regions (Floate et al., 2015; Floate et al., 2017). The highest number of *O. taurus* adults emerged at 24 °C and the species takes 25 days at 30 °C and 105 days at 16 °C for egg-to-adult development (Floate et al., 2015). It has also been shown to perform well at temperatures elevated 2 °C above the diurnally fluctuating temperatures from naturalised locations in eastern Australia (Holley & Andrew, 2020). *Onthophagus taurus* has low and high growth limiting soil moisture of 10% and 120%; and the lower and upper optimal soil moisture of 35% and 80% indicating that this species has a wider soil moisture adaptability from air dry to stagnant water conditions (Floate et al., 2015; Floate et al., 2017). Various adaptive processes and fitness strategies towards colder, humid and extremely dry climate and soil conditions suggest future niche expansion beyond its realised range (Silva et al., 2016).

### Euoniticellus intermedius

*Euoniticellus intermedius* (Fig. 1) is a widespread species in Africa and is one of the popular species in redistribution programmes (Bornemissza, 1976; Tyndale-Biscoe, 1996 ). It was first introduced into Australia from South Africa in 1971 (Bornemissza, 1976). It was also one of the fast-spreading and rapidly established species in the very beginning of the introduction programme in Australia (Bornemissza, 1976; Bornemissza, 1979; Doube et al., 1991). *E. intermedius* was introduced into the USA from Australia, being released into Hawaii in 1974 (Fincher, 1986). Later, *E. intermedius* was redistributed from Hawaii into other US states including California, Texas and Georgia (Anderson & Loomis, 1978; Blume, 1984; Fincher, 1986). Later it entered and spread to Mexico (De Oca & Halffter, 1998; Pablo-Cea et al., 2017). In Mexico, *E. intermedius* is widespread in arid areas, from 0 to 2600 m ASL in lower to higher temperature regions. It is widespread in habitats ranging from open grasslands to closed pine forests (De Oca & Halffter, 1998). The present distribution and spread (Fig. 2) show *E. intermedius* is an invasive species.

*Euoniticellus intermedius* body colour varies from light brown to tan with darker thorax in young adults (Blume, 1984). It is sexually dimorphic with males larger than females (Pomfret & Knell, 2006). Females are hornless and males are horned, and horn size is a fighting morphological trait and is a predictor of victory in male fights and physical performance (Lailvaux et al., 2005; Pomfret & Knell, 2006).

At lower soil moisture regimes, *E. intermedius* produces a high number of brood balls (Barkhouse & Ridsdill-Smith, 1986). An individual *E. intermedius* female lays 120 eggs in her lifetime. A single egg is laid in a brood ball, and a nest comprises a maximum of eight broods (Tyndale-Biscoe & Watson, 1977). The female lays an egg inside the brood ball on top of an inner layer of maternal secretions which is partially digested dung and contributes microbial community (Shukla et al., 2016) that improves the survival fitness of larvae (Byrne, Watkins & Bouwer, 2013). Females can resorb extra ovarian oocytes when the laying environment is hostile (Tyndale-Biscoe & Watson, 1977), which is a rare phenomenon in insects (Moore & Attisano, 2011). Adults generally survive for 45 days and the higher fecundity and short life span of this species usually result in sudden population outbreaks (Blume, 1984).

*Euoniticellus intermedius* is well adapted to dry-arid conditions and upper-temperature extremes (De Oca & Halffter, 1998; Hemmings, 2018; Lobo, 1996). It can nest, hatch, emerge and use dry faeces even in drought conditions and can consistently produce high numbers of broods in soils below wilting points (<3.5% soil moisture) (Barkhouse & Ridsdill-Smith, 1986). Females place brood balls throughout the available soil in clumps that prevent collapse of brood balls (Barkhouse & Ridsdill-Smith, 1986). They make a larger brood chamber and lay a single egg towards the end of the burrow: a strategy to protect eggs from desiccation (Blume, 1984). *E. intermedius* can tolerate extremes of temperatures between 8.2 °C (CT_min_) and 49.8 °C (CT_max_), respectively (Hemmings, 2018). It is a species with a broader thermal window and performs well across a wide range of temperatures and thus avoids competition with other species (Hemmings, 2018). *E. intermedius* can also perform well in a range of life-history traits (such as dung burial, brood production and egg-laying), when adult stages are exposed throughout at 2 °C and 4 °C above the diurnally fluctuating temperatures (Holley & Andrew, 2019). As compared to other dung beetle species, used in redistribution, *E. intermedius* is highly resistant to agrochemicals, such as *Cypermethrin*, with no demonstrated detrimental effects on its survival and reproduction (Kryger et al., 2006).

The fact that *E. intermedius* has a diel activity niche and is active during the afternoon (1400–1700 h) when no or few other dung beetle species are active which reduces temporal competition (Blume, 1984). As compared to other introduced dung beetle species (e.g. *D. gazella*, *O. binodis* and *E. fulvus*), *E. intermedius* is best adapted to open and sunny habitats and also to tropical deciduous forest and semiarid shrubland (De Oca & Halffter, 1998). The distribution patterns and adaptation strategies of *E. intermedius* present it as a successful invader and a potential invasive species.

### Aphodius fimetarius

*Aphodius fimetarius* (Fig. 1) is a European-Asiatic species widespread and abundant in temperate zones of Palaearctic and Oriental regions (Hanski, 1980, 1991; Miraldo et al., 2014). It is an accidental introduction to Australia (Stebnicka, 2009), New Zealand (Stebnicka, 2001), the USA (Horn, 1887) and Canada (Floate, 2011). It is a complex of sibling species that makes it difficult to identify by morphological characteristics (Miraldo et al., 2014; Stebnicka, 2001; Stebnicka & Howden, 1995). It is only one species in the genus *Aphodius* which is distributed across all continents except Antarctica (Fig. 2).

*Aphodius fimetarius* is one of the largest species of genus *Aphodius* (Stebnicka, 2009). It has variations in colour and morphology, but resembles other *Aphodius* species (Angus, Wilson & Krell, 2012; Miraldo et al., 2014). For example, *A. fimetarius* and *A. pedellus* are morphologically similar, but they display distinctly different karyotypes (Miraldo et al., 2014). There are only small morphological differences in the endophallus, head shape of males, and the pronotal punctation of females in the species (Miraldo et al., 2014). *A. fimetarius* is a cosmopolitan dweller species and has enormous reproductive potential among the Aphodiinae (Miraldo et al., 2014). The female lays 100 or more eggs in her lifetime. The adults occur in hundreds in a single dung pat and are found more concentrated in older dung pats (Gittings & Giller, 1997). The adults mate and the female can lay eggs in very old dung pats (Holter, 1982). *A. fimetarius* produces two generations a year, which is rare in other *Aphodius* species and keeps it active in cattle dung pats around all grazing seasons (Gittings & Giller, 1997). This bimodal seasonality is observed during spring with the emergence of overwintered adults and during autumn, with the emergence of adults developed from spring laid eggs (Floate, 2011). However, north or south-facing habitats and soil types affect the timing of the emergence of overwintering individuals (Vessby & Wiktelius, 2003). Females lay eggs singly in the crust, and the larvae develop in dung, but pupation can take place in either the dung and or in the soil. The eggs, third instar larvae, prepupae and adults can overwinter (Gittings & Giller, 1997).

*Aphodius fimetarius* has a critical thermal minimum (CT_min_ ) of −2.51 °C and a critical thermal maximum (CT_max_) of 44.2 °C (Hemmings, 2018). Thus the species can perform over a broad temperature range. The lower metabolic rate when exposed to downward ramping temperatures and higher metabolic rate when exposed to upward ramping temperatures shows *A. fimetarius* is stressed more in higher temperatures (Hemmings, 2018). The lower metabolic rate at downward ramping temperatures and tolerance to lower temperatures (CT_min_ = −2.51 °C) indicate that *A. fimetarius* is a cold-tolerant species. Its low-temperature niche (Hemmings, 2018) and largest body size among the Aphodine dwellers (Stebnicka, 2009) makes it competitively superior as compared to other dung dweller species.

The native European range of *A. fimetarius* shows it can thrive well in harsh environmental and scarce resource conditions. *A. fimetarius* can excavate, shred and break up sizeable old dung pats in cold, dry seasons (Bornemissza, 1979). It also avoids competition with tunnellers and rollers by breeding during times when they are not active or by inhabiting dung that is unsuitable for them. *A. fimetarius* also shows cryptic complexities and high genetic variation within the species (Miraldo et al., 2014). The various adaptation traits make it a successful invader and present it as a potentially invasive dung beetle species.

## Attributes of Invasion and Invasive Species

*Digitonthophagus gazella*, *O. taurus, E. intermedius*, and *A. fimetarius* are the most dominant dung beetle species in grasslands since one or more of the species comprise 50–99% of total dung beetle abundance in invaded ecosystems (Bornemissza, 1976; Filho et al., 2018; Fincher, Stewart & Hunter, 1983; Floate & Kadiri, 2013; Miraldo et al., 2014; Silva et al., 2016; Young, 2007). *D. gazella* and *E. intermedius* in Australia (Bornemissza, 1976) and *D. gazella* and *O. taurus* in the USA (Fincher, Stewart & Hunter, 1983) were spread rapidly within a few years of introduction. In south-western Australia, *O. taurus* has replaced *O. binodis*-one already established popular introduced species-within four years of its introduction (Ridsdill-Smith & Edwards, 2011). Introduced species are most abundant in grassland habitats, and they comprise on average of nine species in northern Australia and 1–2 species in southern Australia (Ridsdill-Smith & Edwards, 2011). Limited studies on temperate riparian and tropical woodland habitats show no introduced dung beetle species detected in the habitats (Ebert et al., 2019; Gollan et al., 2011), but it needs an extensive study to confirm it further. The availability of surplus dung resources in the novel environments enabled the range expansion of several exotic species (Bohle et al., 2009). The invasive dung beetle species are reported from every continent, except Antarctica, and they have expanded their distribution beyond their native range (Barbero & López-Guerrero, 1992; Lobo & Davis, 1999; Pablo-Cea et al., 2017; Rossini, Vaz-de-Mello & Zunino, 2016; Silva et al., 2016). The distribution range expansion by invasive species has blurred the distinctiveness of dung beetle biogeography (Floate et al., 2017; Medina, 2016; Silva et al., 2016).

Invasive dung beetle species possess unique adaptations similar to other invasive invertebrate and plant species (Comont & Roy, 2011; Davidson, Jennions & Nicotra, 2011; Nyamukondiwa, Kleynhans & Terblanche, 2010; Roy et al., 2008). *D. gazella* and *O. taurus* produce multi-brood nests (Table 1) similar to that of invasive European fire ant (*Myrmica rubra*) (Goodman & Warren II, 2019). Lifetime fecundity of *E. intermedius* and *A. fimetarius* is higher than non-invasive dung beetle species of the same genus (Gittings & Giller, 1997; Johnston et al., 2008). At least twice the fecundity is observed in Asian invasive harlequin ladybeetle than in its native counterparts (Michie et al., 2010). Invasive dung beetle species are small-bodied, short-living, produce multiple progenies during several breeding episodes, consume or bury large amounts of dung relative to their body size and have a unique method of brood care (Table 1). Invasive species show greater plasticity across a range of morphological, biological, physiological and fitness traits than in the co-occurring non-invasive species (Davidson, Jennions & Nicotra, 2011). *D. gazella* and *O. taurus*, *E. intermedius* and *A. fimetarius* also show adaptation and fitness traits (Table 1), enabling them to be invasive.

**Table 1:** Life history traits and other characteristics of four invasive dung beetle species.

Characteristics	*Digitonthophagus gazella*	*Onthophagus taurus*	*Euoniticellus intermedius*	*Aphodius fimetarius*
Body colour (melanism)	Brown to dark brown	Black to reddish–brown	Light brown to tan	Half black and half orange–red
Diel activity	Crepuscular	Diurnal	Diurnal	Diurnal
Original distribution	Afro-Asia	Europe	Africa	Europe-Asia
Redistribution type	Deliberate/accidental	Deliberate/accidental	Deliberate/accidental	Accidental
Functional guild	Small tunneller	Small tunneller	Small tunneller	Small dweller
Body length	10–13 mm	5–11 mm	7–9 mm	5–8 mm
Body live and (dry) weight	151 (41) mg	93 (15) mg	53 (8) mg	25 (7) mg
Eggs to adult development (days)	28	30	28	67
Overwintering stages	Larvae and adults	Larvae	Larvae and adults	Eggs, larvae and adults
Generations per year	5	5	6	2
Longevity/adult life span	60 days	56 days	45 days	55 days
Brood ball burial/nest depth	25–35 cm	6–24 cm	0–15 cm	Not applicable
No of eggs per brood ball	Single	Single	Single	Singly in crust
Brood balls in nests	Separate	Separate	Separate	Not applicable
Broods per nest	45	8	10	Not applicable
Life time fecundity	90	Unknown	120	100
Critical temperature minimum and maximum (CTmin and CTmax)	Unknown	Unknown	8.28 °C and 49.80 °C	−2.51 °C and 44.20 °C
Metabolism (V̇CO_2_ ml h^−1^) at CTmin and CTmax	Unknown	Unknown	0.73 ± 0.19 and 2.94 ± 0.09	1.37 ± 0.18 and 2.95 ± 0.09
Degree-days per generation (above limiting low temperature)	209	210	Unknown	Unknown
Growth limiting low and high temperatures (°C)	18 °C and 34 °C	14 °C and 32 °C	Unknown	Unknown
Lower and upper optimal temperatures (°C)	25 °C and 30 °C	16 °C and 23 °C	Unknown	Unknown
Growth limiting low and high soil moistures (%)	5 and 200	10 and 120	Unknown	Unknown
Lower and upper optimal soil moistures (%)	35 and 100	10 and 80	Unknown	Unknown
Dispersal	50–800 km year ^−1^	300 km year^−1^	50–480 km year^−1^	Unknown
Global geographical coverage	15 million km^2^	Unknown	12 million km^2^	Unknown
Climatic niche	Tropical to subtropical	Sub-tropical to temperate	Tropical and subtropical	Temperate or winter
Habitats	Open pasture	Open pasture	Open pasture and forests	Open pasture
Dung feeding	Generalist	Generalist	Generalist	Generalist

**Note:**

Sources: Cambefort (1991), De Oca & Halffter (1998), Doube et al. (1991), Fincher, Stewart & Hunter (1983), Floate et al. (2015), Floate et al. (2017), Hemmings (2018), and Rougon & Rougon (1991).

*E. intermedius* and *A. fimetarius* multiply and thrive well in disturbed habitats and with remnant dung resources (Barkhouse & Ridsdill-Smith, 1986). Others have also mentioned that invasive species colonise disturbed habitats, and invade weak local communities which lack competitors (Braga et al., 2013; Comont & Roy, 2011). Similarly, red imported fire ant, *Solenopsis invicta* Burren, a notorious invasive species, is a disturbance specialist and is reportedly invasive in human-modified habitats (King & Tschinkel, 2008). The invasive dung beetle species are open habitat specialists and most abundant in open pasturelands (Barbero & López-Guerrero, 1992; Fincher & Woodruff, 1975), but *D. gazella* has been reported from closed forest habitats (De Oca & Halffter, 1998). Climate change, harsh and fluctuating weather conditions, and decline and degradation of biodiversity favour invasive species (Coristine & Kerr, 2011; Floate & Kadiri, 2013).

*Digitonthophagus gazella* is genetically diverse, with several biotypes that can adapt to different climatic conditions (Bornemissza, 1979; Floate et al., 2017). Most of the invasive species are prone to genetic changes and have developed multiple phenotypes in comparison to co-occurring resident species (Davidson, Jennions & Nicotra, 2011; Ricciardi et al., 2017). Introduced invertebrate predators and microbial pest control agents are predicted to develop more biotypes or strains than the co-occurring native species (Comont & Roy, 2011; Roy & Wajnberg, 2007). The genetic variation happens through microevolution during range expansion, subsequent extinctions, and relocations of species (Michie et al., 2010).

## Impacts of Invasive Dung Beetles

Invasive dung beetles have been shown to have detrimental effects on native dung beetles through reduced native species assemblage diversity, reduced populations abundance and sometimes local extinctions (De Oca & Halffter, 1998; Filho et al., 2018; Genier & Davis, 2017; Kohlmann, 1994; Medina, 2016; Vidaurre, Noriega & Ledezma, 2008). They can outcompete the native dung beetle species that overlaps their nesting behaviour and phenology (Filho et al., 2018). The invasions have changed the ratio of functional guilds—namely rollers, tunnellers and dwellers—with the appearance of a new dweller community (Filho et al., 2018; Gordon, 1983). Native fauna extinctions and detrimental effects on local assemblages have created an ecological crisis in the invaded ecosystems (Blanco et al., 2013; Filho et al., 2018). Thus, the invasion has threatened the future, quality and sustainability of the invaded ecosystem (Floate et al., 2017; Manning & Cutler, 2018; Silva et al., 2016). The impacts could be more severe in islands as the native species will have no area to extend or expand (Kumar et al., 2015). Invasive dung beetles have provided evidence in support of the idea that the altered ecosystems might not be novel and inevitable in the long-run (Belnap et al., 2012; Boivin et al., 2016).

The impact of invasion is context-dependent, and as in other invaded ecosystems, the heterogeneity of habitats, dung beetles assemblage structure and lack of pre-invasion data can make impact assessment hard to quantify (Gutierrez, 2017; Myers & Cory, 2017; O’Hea, Kirwan & Finn, 2010; Tylianakis et al., 2008). Substantive quantitative data is required in order to assess the impacts of invasive dung beetles on ecosystem services (Hulme & Vilà, 2017; Naidoo et al., 2008; Soliveres et al., 2016). Robust empirical evidence is necessary for a broader understanding of the impact of exotic species on native fauna and assemblages (Foxcroft, Pickett & Cadenasso, 2011). Therefore, the persistence of invasive dung beetle species and their long-term numerical abundance in the habitats can perhaps be considered positive if they provide sustainable ecosystem services (Belnap et al., 2012).

Biological invasions are commonplace in nature (Epanchin-Niell, 2017). However, not every introduced species became invasive, as they have to surmount the complexities of geographical, reproductive and dispersal barriers (Foxcroft, Pickett & Cadenasso, 2011). On all spatial scales, species ranges change continuously, which makes assemblage structuring a complicated process (Gilchrist & Lee, 2007). Exotic species colonise particular niches in the degraded land or poor unoccupied habitats and maintain their self-sustaining populations (Ricciardi et al., 2017). An ecosystem is ideal if native and exotic species are interacting and sharing the resources without a negative impact on the environment and local assemblages (Gollan et al., 2011).

Redistribution of exotic species increases invasion risk as the exotic species may adapt better to a novel environment than the co-occurring native species (Caño et al., 2008). Climate change is a current abiotic factor that is changing the distributions of native species (Tingley et al., 2014) and they might expand their distribution range and can become invasive; but the impact is generally less severe as they share a common physical environment and a long evolutionary history with the co-occurring species (Ricciardi et al., 2017). Contrary to this, exotic species perhaps: may have substantial negative impacts and are likely to be more invasive than native species (Bos et al., 2008). Dung beetles have been redistributed as biocontrol agents (BCAs) of dung (Bornemissza, 1970; Macqueen & Beirne, 1975). There is evidence that a large number of deliberately introduced BCAs have caused negative impacts on biodiversity and invaded ecosystems (Gutierrez, 2017; Kumar et al., 2015; Ricciardi et al., 2017). Biocontrol agents that have become invasive include the Asian harlequin ladybeetle in the UK (Comont & Roy, 2011), the introduced exotic wasp, *Vespula pensylvanica* (Saussure), in Hawaii (Funasaki et al., 1988) and the common wasp, *Vespula Vulgaris* (Linnaeus), in New Zealand (Elliott et al., 2010).

## Recommendations and Conclusions

Australian and North American dung beetle redistribution programmes followed strict quarantine to prevent the spread of pests and diseases (Bornemissza, 1976), but invasion potentials of the species were never considered (Dymock, 1993). Future redistribution programmes need to consider the positive and negative impacts on ecology, environment and human health of the populations of species being moved (Bishop et al., 2005; Dymock, 1993; Mackereth et al., 2013; Myers & Cory, 2017). The impact of exotic dung beetles on non-target organisms such as earthworms, other terrestrial invertebrates and native dung dwelling fauna are equally important (De Nardo et al., 2006; Dillon et al., 2012). Moreover, the exotic species might undergo evolutionary changes in the novel environment, but potential change and prediction of how this change will happen is impossible to predict without detailed ecological information (Brandenburger et al., 2019; Michie et al., 2010). Therefore, careful assessment of the ecological functions provided by native and naturalised dung beetle assemblages is essential to avoid unnecessary additional introductions (Funasaki et al., 1988; Nakao & Funasaki, 1979).

Furthermore, baseline and periodic monitoring of native dung beetle assemblages before the introduction of exotic species are vital for designing dung beetle redistribution programmes (Funasaki et al., 1988). The consequences of invasive dung beetle species in unintended regions in South American countries signifies a need for a coordinated intercountry panel to discuss any negative implications of redistributions programmes to the bordering countries (Hulme & Vilà, 2017; Ricciardi et al., 2017). A critical review of Australasian and Northern American dung beetle redistribution programmes might give insights for future dung beetle redistributions. The science-policy interface of the Intergovernmental Platform on Biodiversity and Ecosystem Services (IPBES) might be a suitable forum for the further discussion of risks and benefits of dung beetle redistribution in countries and the regions (Epanchin-Niell, 2017).

A pre-introduction and a post-establishment quantitative database of native and introduced dung beetle species are essential to assess any impacts on the recipient ecosystem. The invaded ecosystems will be fascinating natural experiments for future ecological studies if a baseline and natural history database of native and introduced dung beetle communities are maintained. A more in-depth understanding of adaptation attributes of introduced dung beetle species would be useful in the prediction of invasiveness of the species.

## Additional Information and Declarations

### Data Availability

The following information was supplied regarding data availability: This literature review does not have raw data or code.
